# Multiscale mechanical characterisation of the craniofacial system under external forces

**DOI:** 10.1007/s10237-023-01799-y

**Published:** 2024-01-13

**Authors:** Marius Didziokas, Dominic Jones, Ali Alazmani, Miranda Steacy, Erwin Pauws, Mehran Moazen

**Affiliations:** 1 Department of Mechanical Engineering, University College London, London, UK; 2 School of Mechanical Engineering, University of Leeds, Leeds, UK; 3 Developmental Biology and Cancer Research and Teaching Department, UCL Great Ormond Street Institute of Child Health, University College London, London, UK

**Keywords:** Craniofacial system, Biomechanics, Suture, Craniosynostosis, Mechanobiology

## Abstract

Premature fusion of craniofacial joints, i.e. sutures, is a major clinical condition. This condition affects children and often requires numerous invasive surgeries to correct. Minimally invasive external loading of the skull has shown some success in achieving therapeutic effects in a mouse model of this condition, promising a new non-invasive treatment approach. However, our fundamental understanding of the level of deformation that such loading has induced across the sutures, leading to the effects observed is severely limited, yet crucial for its scalability. We carried out a series of multiscale characterisations of the loading effects on normal and craniosynostotic mice, in a series of in vivo and ex vivo studies. This involved developing a custom loading setup as well as software for its control and a novel in situ CT strain estimation approach following the principles of digital volume correlation. Our findings highlight that this treatment may disrupt bone formation across the sutures through plastic deformation of the treated suture. The level of permanent deformations observed across the coronal suture after loading corresponded well with the apparent strain that was estimated. This work provides invaluable insight into the level of mechanical forces that may prevent early fusion of cranial joints during the minimally invasive treatment cycle and will help the clinical translation of the treatment approach to humans.

**Supplementary Information:**

The online version contains supplementary material available at 10.1007/s10237-023-01799-y.

## Introduction

The neonatal mammalian skull roof consists of five bones that are joined together along their edges by soft fibrous tissues called cranial sutures. Sutures facilitate birth and accommodate postnatal brain growth. (Herring 1972; Opperman 2000; Richtsmeier and Flaherty 2013; Liang et al. 2023) Once the brain has reached its maximum size, visible gaps at the sutures are reduced to micrometre gaps where sutures have differentiated to bone. Our fundamental understanding of the mechanobiology of the craniofacial system, especially during the development of the craniofacial system, is limited. (Wang and Mao 2002; Mao 2002, 2003; Kopher and Mao 2003; Peptan et al. 2008; Takeshita et al. 2017) For example, we still do not know what level of mechanical strain this system experiences during ab/normal development and its repair or how it will respond to changes in its local mechanical environment. (Tanaka et al. 2000; Kopher et al. 2003; Oppenheimer et al. 2012) This has impacted the treatment of various craniofacial conditions, such as large calvarial defects in adults or congenital conditions in infants.

Craniosynostosis (CS) is a major condition affecting 1 in 2000 births, with its prevalence increasing 2–3 times in recent years. (van der Meulen et al. 2009; Johnson and Wilkie 2011; Cornelissen et al. 2016; Tønne et al. 2020) CS is caused by early fusion of the sutures. While craniofacial surgeons have been developing various techniques to advance the treatment of this condition (David and Sheen 1990; Jimenez and Barone 1998; Rahimov et al. 2016; Delye et al. 2018; Breakey et al. 2021) molecular biologists have been unravelling its underlying genetics and have developed various mouse models presenting the condition. (Rice et al. 2003; Eswarakumar et al. 2004; Ishii et al. 2015; Flaherty et al. 2016; Katsianou et al. 2016; Merkuri and Fish 2019; Lee et al. 2019) The Crouzon mouse model, type *Fgfr2*^*C342Y/*+^, was developed in 2004, following the discovery of the genes responsible for early fusion of the coronal suture (joining the parietal and frontal bones) in humans. (Oldridge et al. 1995) In this well-established mouse model, the coronal sutures are primarily affected, causing a short and domed head shape. (Perlyn et al. 2006; Martínez-Abadías et al. 2013; Liu et al. 2013) The coronal suture in the Crouzon mouse typically starts to obliterate/fuse at embryonic stages (E18.5); the rate of this fusion varies between individuals, and full closure is typically achieved at P20. The coronal sutures do not fuse in the wild-type mouse. (Eswarakumar et al. 2004; Peskett et al. 2017).

**Fig. 1:**
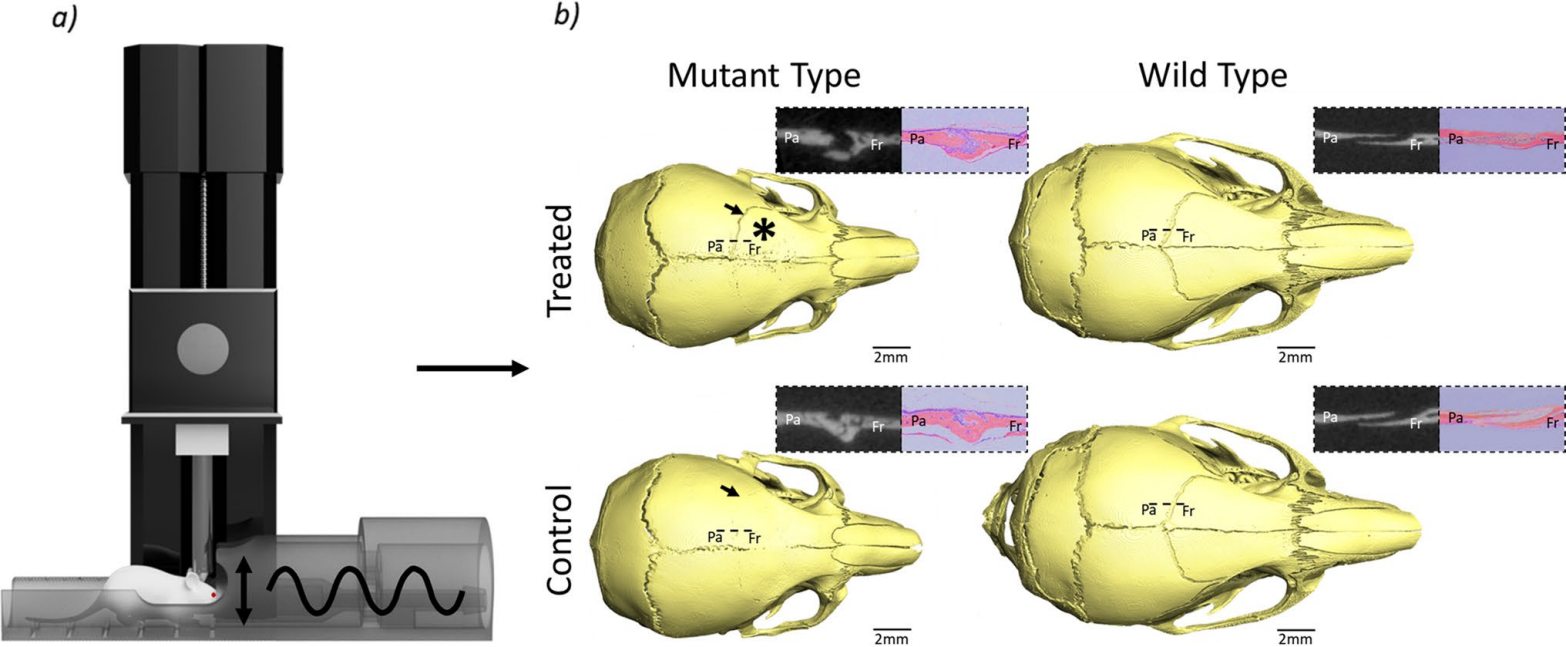
Effects of external cyclic in vivo frontal bone loading in the dorsoventral direction on coronal suture patency in Crouzon and wild-type mice. **a** Loading setup schematic and **b** coronal suture patency results at P21 including CT and histological slices through the coronal suture, * indicates the approximate loading location and arrows highlight the left coronal suture. Interpreted from Moazen et al. (2022)

Recently, Moazen et al. (2022) discovered that through minimally invasive cyclic unilateral loading of the frontal bone in the dorsoventral direction, they could delay the early fusion of the coronal suture (unilaterally) in the Crouzon mouse and may help restore its skull morphology. Their treatment started from postnatal day 7 (P7) and was carried out for a total of 10 days with an external force of 0.1 N at 1 Hz for 10 min per day (from P7-P11 and then P14-P18 and animals were culled at P21). (Fig. 1) However, it remains unclear how their treatment approach altered the mechanics of the skull or precisely what level of mechanical strain was induced across the coronal suture that led to the aforementioned phenotypic changes. Such data are crucial to advance our understanding of the mechanobiology of the craniofacial system. Nonetheless, the multiscale nature of such studies and the presence of various soft and hard tissues make such analysis challenging.

The overall aim of this study was to characterise the impact of external forces on the mechanics of the craniofacial system in wild-type and Crouzon mice. A series of multiscale analyses were performed analogously to the previous study by Moazen et al. (2022) to quantify the level of mechanical strain induced across the calvarial sutures. The specific objectives of this study were to quantify the impact of calvarial loading on the (1) whole head; (2) coronal suture size; (3) level of mechanical strain induced across the calvarial sutures.

## Materials and methods

**Fig. 2:**
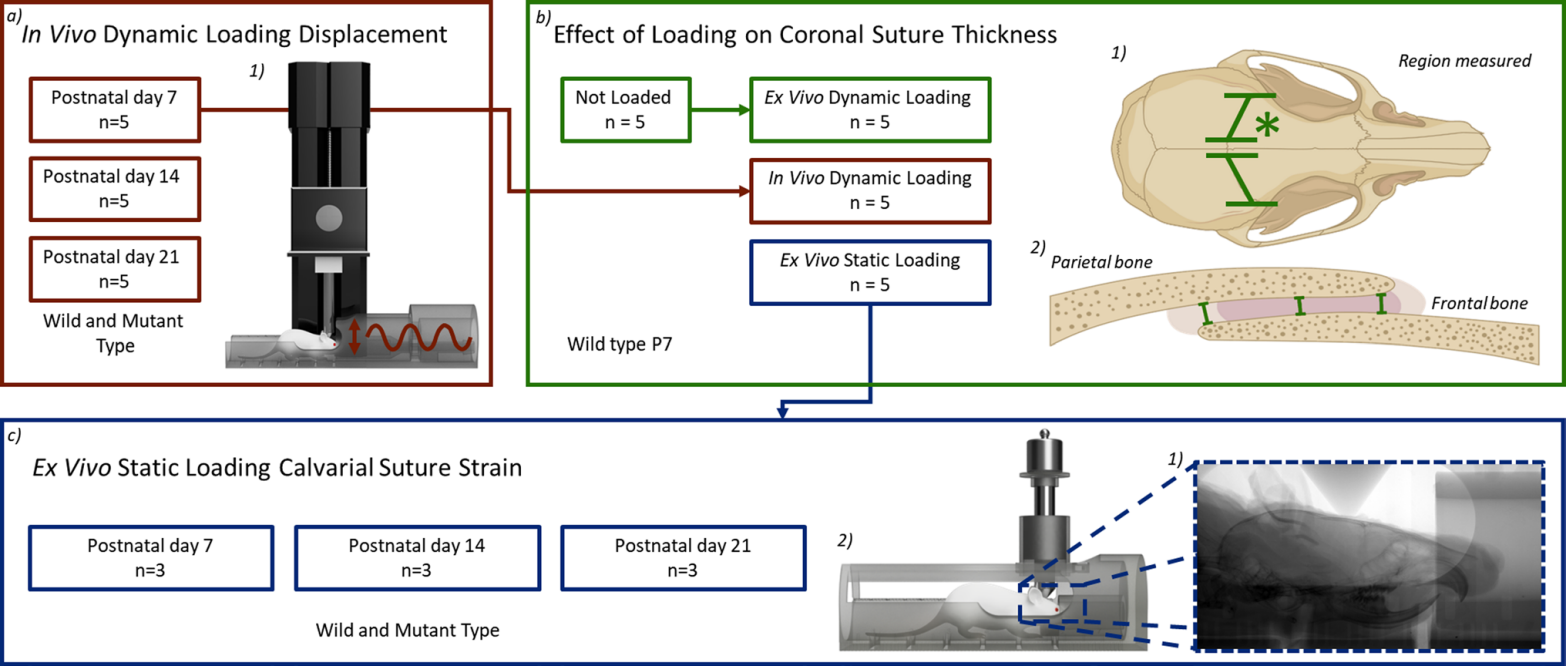
An overview of various experiments and analyses performed in this chapter. This includes the type of experiments conducted and the number and type of animals used for each experiment. **a** In vivo dynamic loading displacement characterisation 1) dynamic loading setup schematic, **b** suture thickness after static and dynamic loading 1) region measured with * denoting the loading location for all experiments, 2) measurements taken across the coronal suture and **c** ex vivo static loading suture strain quantification 1) radiography image during loading, 2) static loading setup schematic

In the first step, a series of in vivo experiments were carried out on wild-type and Crouzon *Fgfr2*^*C342Y/*+^ mice at specific ages (P7, P14 and P21). These experiments were force-controlled, and the loading tip displacement was quantified as a measure of the whole head displacement. In the second step, a series of *in/*ex vivo dynamic and static loading experiments were carried out (using P7 WT specimens) to quantify the level of loading tip displacement and the lasting size change across the coronal suture (via quantification of suture thickness changes) as a result of the aforementioned tests. This investigation enabled us to understand to what extent an ex vivo static loading scenario on fresh cadavers may differ from an in vivo dynamic loading experiment. In the third step, a series of ex vivo static loading experiments were carried out (at P7, P14 and P21), and a novel algorithm was developed to quantify the level of mechanical strain induced across the calvarial sutures. This analysis was carried out ex vivo and under static loading due to the high-quality micro-CT images required. The scans lasted 2 h, and the duration of anaesthesia as well as the radiation dose prohibited these experiments in vivo. Note that in all cases, the left frontal bone was loaded in the dorsoventral direction (see Fig. 2).

### Specimens

Wild-type and mutant mice were investigated; for the latter, *Fgfr2*^*C342Y/*+^ Crouzon mouse model was used. These were derived by the European Mouse Mutant Archive (EMMA) at MRC Harwell, as described by Peskett et al. (2017). Ex vivo studies were carried out on fresh, refrigerated (4°C) cadavers, i.e. the analyses were carried out within four days of the animals being culled.

Figure 2 summarises the number of animals used per different experiments carried out in this study. In total, 63 animals were used. For the in vivo dynamic loading, 5 animals were used per group (P7 WT, P14 WT, P21 WT and P7 MT, P14 MT, P21 MT). For the lasting suture thickness analysis, 5 animals were used per group (P7 WT not loaded/ex vivo dynamic loading, P7 WT in vivo dynamic loading and P7 WT *ex* vivo static loading). Lastly, for the suture strain analysis during ex vivo static loading, 3 animals were used per group (P7 WT, P14 WT, P21 WT and P7 MT, P14 MT, P21 MT).

Note all animal experiments were approved by the UK Home Office and performed as part of a Project License (number: 70/8817) under the UK Animals (Scientific Procedures) Act 1986. Animal procedures complied with the ARRIVE guidelines and were performed under the supervision of UCL Biological Services.

### Loading setups

*Dynamic loading:* was applied using a custom-built test system (see Fig. 2b). Here, an actuator (Newmark Systems, Inc: res. 0.04 µm with Maximum velocity of 25 mm/sec) and a force sensor (GSO Series, Transducer Techniques: res. 0.01 N with 1.5 N capacity) were used with a custom developed LabVIEW programme (National Instruments Corp, Austin, TX, USA). The dynamic loading was force-controlled and conducted following the protocols used by Moazen et al. (2022). The mice were loaded for 10 min at 1 Hz with 0.1 N maximum load.

*Static loading:* was applied using a custom-built setup mimicking the dynamic loading setup (Fig. 2c) to ensure the loading position and force remained as close as possible to the in vivo setup. Here, the animals were placed in a simplified anaesthetic tube to preserve the same points of contact during loading, and a 10 g (0.1 N) weight was placed on a plunger with the same diameter tip as in the dynamically loaded specimens. The static loading was conducted in a micro-CT scanner (XT H 225ST, Nikon, Herts., UK), and the specimens were scanned in place before and during the loading. All the CT scans were taken at 90 kV for 2 h with a voxel size of 9.5 µm.

A load-relaxation test was carried out using the setup inside the micro-CT scanner. Here, the skull was loaded at 0.1 N, and radiography images were taken every 15 s for 180 min. It was found that after 120 min, there was minimum deformation across the skull (**see Supplement 1**). Hence, all static loading experiments inside the CT scanner lasted ca. 240 min with 120 min of relaxation and 120 min of continuous scanning time with the load applied throughout the 240 min time period. A 120-min scan prior to the loading and relaxation was also carried out. This was the major difference compared to dynamic loading and a limitation of this work.

### Measurements

*Loading tip displacement:* Dynamic loading setup had an encoder built into it. Hence, the loading tip’s displacement data during the dynamic loading was measured directly from the system. Here, two parameters were quantified: (1) the maximum displacement of the head and (2) the difference between the maximum and minimum displacement for each oscillation cycle in the last 30 s of the loading. The aforementioned time period was used to reduce the variability in the results, especially as the oscillation max–min values were significantly affected by the animal’s breathing patterns. In the static loading, tip displacement data was measured from the CT data obtained before and during the loading.

*Suture thickness:* Coronal suture thickness was measured using the micro-CT data. The measurements were taken over 250 slices (in the mediolateral plane) on each side (i.e. left and right coronal suture) at three points: the edge of the frontal bone, the midpoint and the edge of the parietal bone in the sagittal plane (Fig. 2a). These results were then averaged for each specimen. Note that coronal suture thickness was measured only in wild-type P7 animals.

*Mechanical strain across the sutures:* A custom programme was written in Python (Python Software Foundation, USA) to calculate the deformation patterns across the bones using the CT data before and after the loading. The programme was based on the digital volume correlation concept. (Buljac et al. 2018; Dall’Ara and Tozzi 2022) Commercially available digital volume correlation algorithms could not be used due to the extremely small thickness of bone when compared to the scan volume that included the whole craniofacial system. (Aggarwal et al. 2009) The displacement data across the bones could then be used to calculate the strains present in the calvarial sutures. Several tests were performed to assess the validity of the programme developed here (see Supplement 2).

**Fig. 3:**
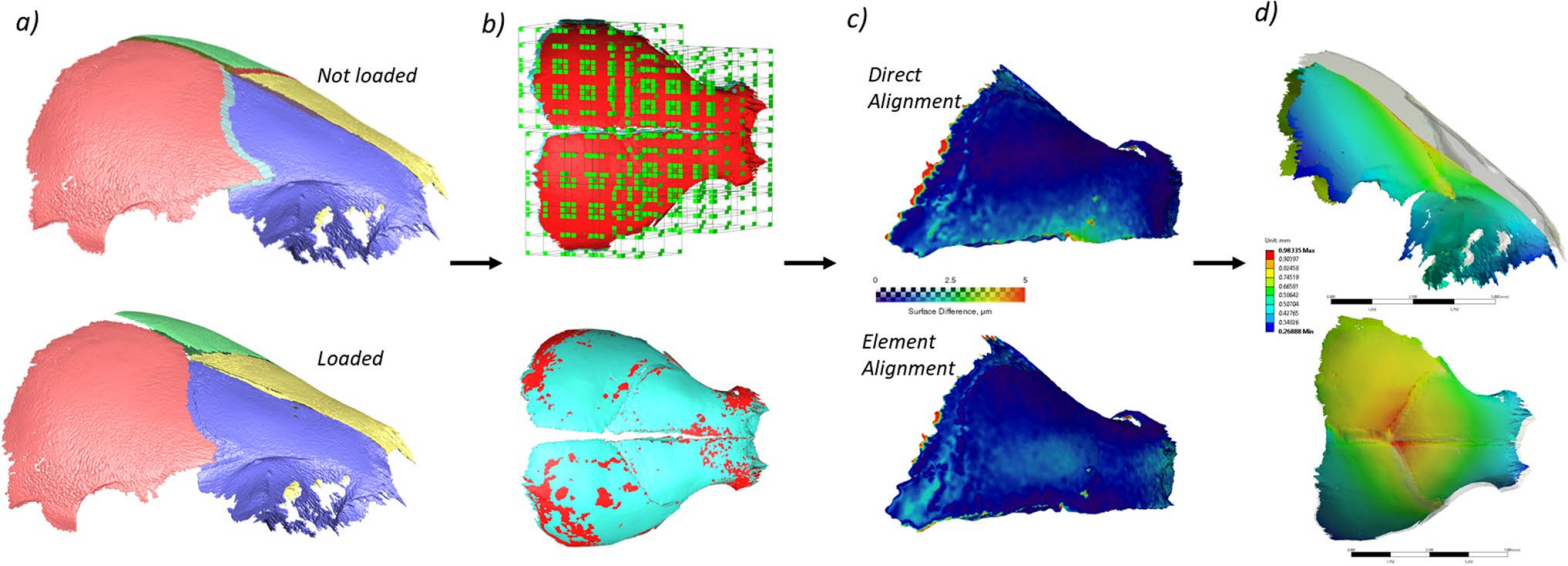
Strain estimation during calvarial loading methodology. **a** Not loaded and loaded states of the same individual, **b** separation of the surfaces to square elements, **c** comparison between direct alignment of surfaces and element separated alignment of surfaces and **d** visualisation of displacement and calculation of strain field using FE (ANSYS)

The overall workflow for the proposed approach here is summarised in Fig. 3. In brief, firstly, the loaded and unloaded scans were segmented using common thresholds (Fig. 3a). This was to minimise variability due to manual segmentation. This was possible because all of the animals were scanned using the same machine with identical scanning parameters. The segmented unloaded scan was then divided into square elements, where each element was rigidly aligned individually to the loaded surface (Fig. 3b). 100 elements per bone were used, as this was found to best capture the deformation. More elements could not be used as the features in each would not be sufficient to confidently align with the deformed body. Figure 3c illustrates the differences in accuracy between direct and element alignment). The displacement at the boundaries between the elements was then smoothed using a centred average for each node 0.25 times the element size to obtain the deformation pattern. The displacement data for each node of the bone surface was then imported into a commercial FE solver (ANSYS, V2022 R2, ANSYS Inc., Canonsburg, PA, USA) as boundary conditions to estimate the strain in the sutures.

It should be noted that (i) while the programme developed here provided an accurate estimation of the strain across the sutures, it overestimated the strain in the calvarial bones; hence, the strain data on the bone is not reported here (see Supplement 2); (ii) a significant limitation of the programme developed here was that the sutures had to be continuous to provide full disarticulation between the bones in the CT data. This was not the case for the coronal suture in the Crouzon mice. Nonetheless, the level of strain in other sutures was quantified.

### Statistical analysis

Statistical analysis was performed in SPSS (IBM SPSS, NY, USA). One-way analysis of variance (ANOVA), with Levene’s test used to test for equal variances. The significance level was set at *p* < 0.05.

## Results

### In vivo *loading displacement (P7, P14 and P21—MT and WT)*

**Fig. 4:**
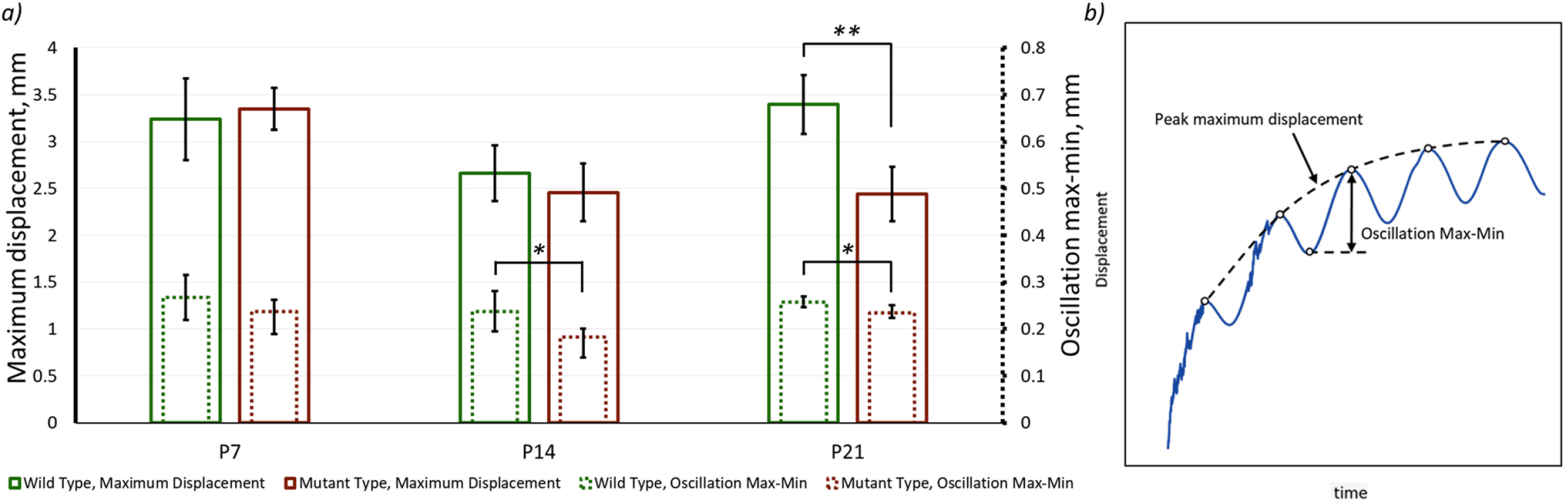
**a** Maximum oscillation displacement (left value bar) and Max–Min oscillation (right value bar) values for the final 30 s of loading comparing wild-type and mutant-type animals. * and ** mark differences between the groups at *p* < 0.05 and *p* < 0.01, respectively and **b** explanation of the values presented in a

The maximum head displacement across all considered cases was in the range of 2.2–3.7 mm, while the displacement within each oscillation (oscillation max–min) was in the range of 0.13–0.32 mm (Fig. 4). It was clear that the maximum displacement was primarily linked with the initial position of the animal in the anaesthetic tube, while the displacement within one oscillation may be reflecting the level of loading applied to the craniofacial system (see Supplement 3 the displacement data over 10 min of loading).

The differences in both recoded displacement quantities were more apparent between the WT and MT mice as the animals became older. There was no significant difference in the displacement quantities between the WT and MT animals at P7. Still, there was a significant difference (p < 0.05) in oscillation displacement between the WT and MT animals at P14 and P21.

### Comparison between the in vivo and ex vivo loading (P7—WT)

**Fig. 5:**
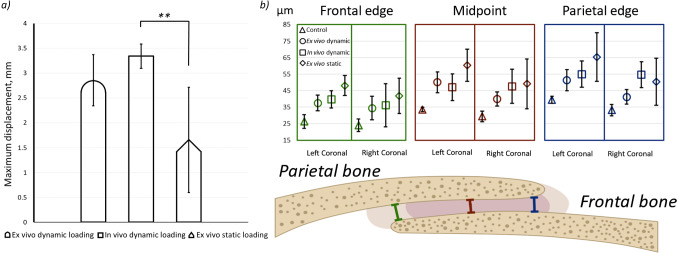
**a **A comparison of the maximum displacement of the loading tip for ex vivo/in vivo dynamic and ex vivo static loading regimes for P7 WT mice. **—*p* < 0.01 and **b** suture thickness as measured after loading at 3 different points in the coronal suture (edge of the frontal bone, midpoint, and edge of the parietal bone) over 250 slices laterally on each side for P7 WT mice after each loading regime

The whole head displacement was compared between the in vivo dynamic, ex vivo dynamic, and ex vivo static loading experiments (Fig. 5a). This data highlighted that the head displacement in the ex vivo static loading scenario was lower than the two other considered loading scenarios. This was statistically significant when comparing the ex vivo static loading vs. in vivo dynamic loading scenarios (1.65 mm ± 1.05 mm vs. 3.34 mm ± 0.25 mm—*p* < 0.01).

In addition to the whole head displacement, suture thickness was also quantified across the aforementioned loading scenarios and compared to an unloaded control group (Fig. 5b). This data highlighted that:

1. Coronal suture thickness across all loading scenarios was significantly larger than the control group (*p* < 0.05). The only exception was at the right coronal suture (i.e. unloaded side) for the in vivo dynamically loaded (*p* = 0.085) at the frontal edge. This suggested a permanent deformation across the sutures under all considered loading scenarios.
2. The coronal suture thickness on the loaded side (left) was slightly higher than on the unloaded side. However, this was not statistically significant when comparing left to right sides’ results within each group.
3. There was no statistically significant difference in the thickness of the coronal suture between the considered loading scenarios. The only exception was the left coronal suture thickness across the frontal edge, where the ex vivo static loading scenario significantly differed from the in vivo dynamic loading (*p* = 0.047). Nonetheless, there was a clear albeit not statistically significant, pattern of higher thickness being measured for the ex vivo static loading compared to the *in/*ex vivo cyclic loading scenarios. For example, the ratio of the coronal suture measured between the mean increase from the control unloaded group were 0.38 ± 0.09, 0.51 ± 0.10 and 0.70 ± 0.10 times the unloaded thickness for the ex vivo dynamically, in vivo dynamically and ex vivo statically loaded groups, respectively.

### Ex vivo static loading—suture strain (P7, P14 and P21 – MT and WT)

**Fig. 6:**
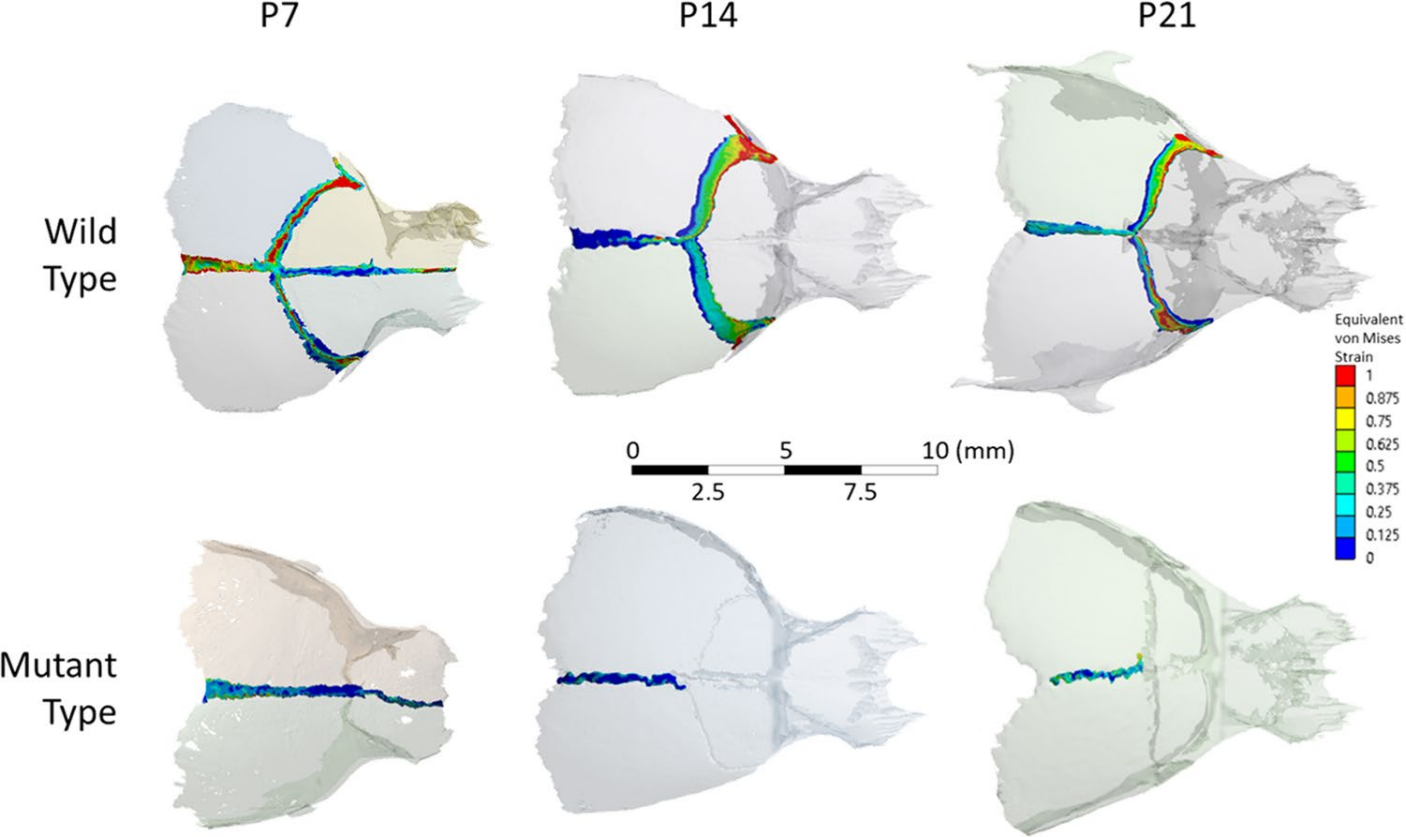
von Mises strain contours for mutant and wild-type specimens at P7, P14, and P21 during ex vivo static loading

**Fig. 7:**
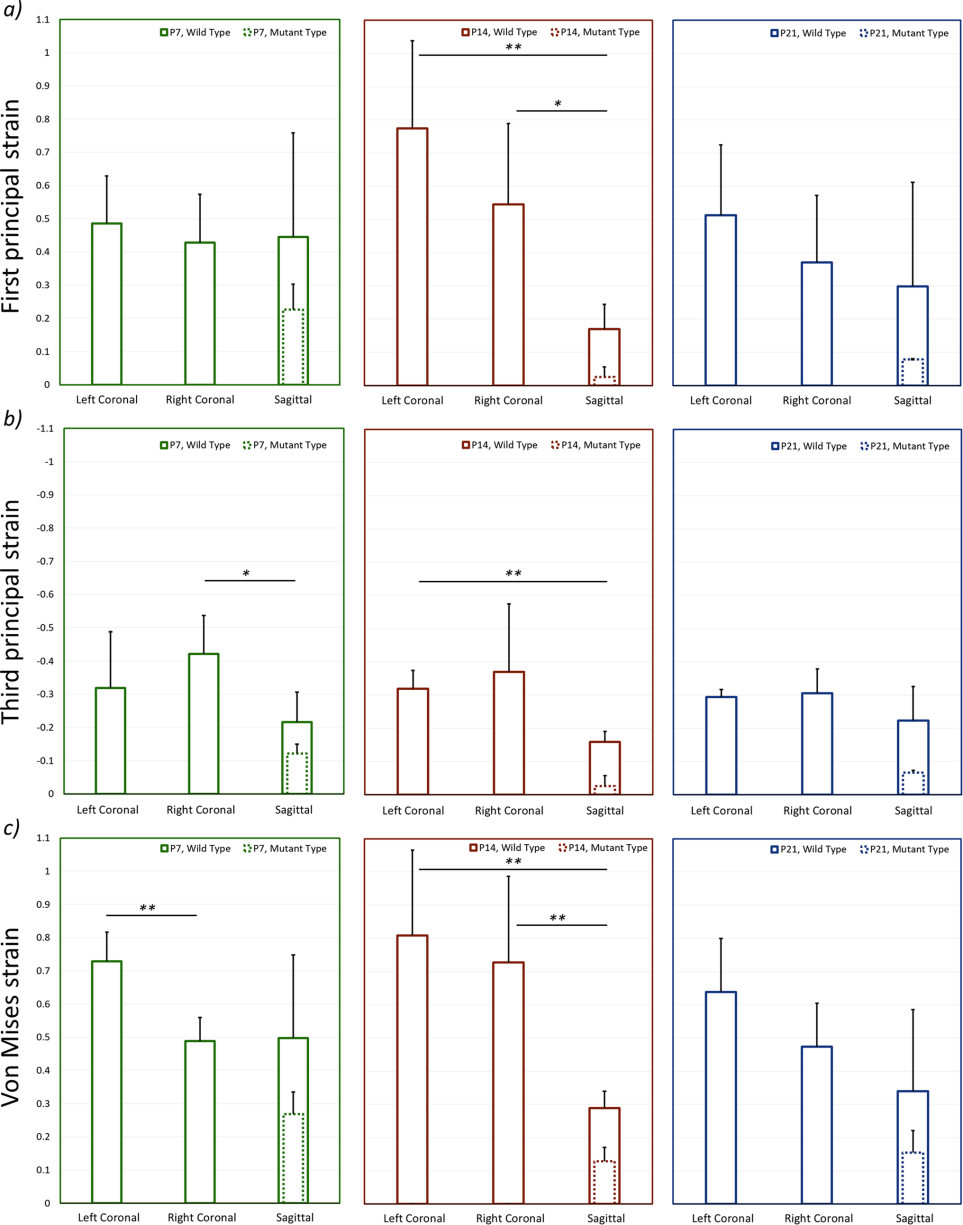
Average strain across each suture for mutant and wild-type specimens at P7, P14, and P21 during ex vivo static loading. **a** First principal strain, **b** third principal strain and **c** von Mises strain. *—*p* < 0.05 and **—*p* < 0.01

The pattern and level of mechanical strain induced across the calvarial sutures as a result of unilateral loading on the frontal bone (at 10 g) are shown in Figs. 6 and 7. Additional data as per individual variation in the three considered specimens for each WT group is included in **Supplement 4**. Overall this data highlighted that:

1. The coronal suture on the loaded side was under higher mechanical strain compared to the unloaded side across the considered ages (e.g. 0.73 ± 0.08 vs. 0.49 ± 0.07 von Mises strain at P7—Fig. 7c). This suture was predominantly loaded under tension across the considered ages, however, was not as clear on the unloaded side, e.g. considering the P7 there was a similar level of 1st and 3rd principal strain across the coronal suture in the unloaded side (Fig. 7a and b at P7).
2. A direct comparison between the WT and MT mice across the coronal suture was not possible; however, considering the sagittal suture, a lower level of strain was recorded in the MT compared to the WT mice across the considered ages (e.g. 0.26 ± 0.07 vs. 0.50 ± 0.25 von Mises strain at P7—Fig. 7c).
3. There was no clear pattern of increase or decrease in the strain level from P7 to P21 across the coronal suture. However, the strain level across the sagittal suture significantly decreased from P7 to P14 in both WT and MT (e.g. 0.50 ± 0.25 vs. 0.29 ± 0.05 for von Mises strain in the WT; *p* < 0.05—Fig. 7c).

## Discussion

A previous study by Moazen et al. (2022) discovered that through external, cyclic, unilateral loading of the frontal bone in the dorsoventral direction, the early fusion of the coronal suture (unilaterally) in the Crouzon mouse (MT) could be delayed. Here, a series of in/ex vivo experiments were carried out to provide insight into the level of mechanical strain induced across the coronal suture in the aforementioned study. Such data are crucial to optimise this approach in mice and scale it up for larger animal models in preparation for potential clinical translation to humans.

Three sets of experiments were carried out to establish the difference between the in vivo dynamic and ex vivo static loading experiments, which were eventually used to quantify the strain level across the calvarial sutures. Initially, the level of whole head movement during the in vivo experiments was quantified at P7, P14 and P21 (Fig. 4). Given that the maximum deformation was expected to occur at P7, this age was then used to compare the effect of in vivo dynamic loading vs. ex vivo dynamic loading and ex vivo static loading (Fig. 5a). These results highlighted that the overall head displacement during the ex vivo static loading was significantly smaller than during in vivo dynamic loading. This likely occurs as the parameter is more likely to have been impacted by the dynamic vs. static nature of these experiments.

The cause of the lower whole head displacement of the ex vivo statically loaded animals compared to their in vivo dynamically loaded counterparts is unclear. One possible explanation may be that the static loading does not allow for the same adjustments to be made during loading that the dynamic allows by removing the load every oscillation. Another possible explanation may be that during dynamic loading more of the load is transferred towards moving the body. The latter is supported by a larger increase in the coronal suture thickness after loading in the ex vivo statically loaded animals.

A larger coronal suture thickness and higher variation in this measurement were observed for the ex vivo static experiments as opposed to the in vivo dynamic loading experiments, albeit not statistically significant. This may be due to the higher loading duration in the ex vivo static experiment, i.e. 4 h to adjust for the relaxation as opposed to 10 min in vivo experiments. It may also be explained by more of the force of the dynamic loading being absorbed by the body, leading to the aforementioned higher whole head displacement.

The suture thickness analysis and the comparison with an unloaded control group at P7 highlighted that: (i) a permanent deformation had occurred in the coronal suture as a result of 10 min in vivo dynamic loading; (ii) the level of mechanical strain induced at the coronal sutures during the in vivo loading was likely at least 0.51 ± 0.10 based on the permanent mean thickness increase across the coronal suture after the loading observed in data presented in Fig. 5b; (iii) ex vivo static loading experiments may overestimate the level of minimum mechanical strain induced across the coronal suture during dynamic loading. A higher estimated strain of at least 0.70 ± 0.10 based on the mean increase in thickness across the coronal suture was observed (Fig. 5b).

The ex vivo and *in* vivo dynamically loaded animals showed a comparable increase in the coronal suture thickness with no statistically significant differences. Consequently, the increase in the suture thickness cannot be a biological response of the suture to the loads instead, the sutures are likely experiencing plastic deformation. This suggests that the increase in suture thickness during the loading is at least equal to the lasting increase if not higher. Thus, the minimum strain estimations presented here were obtained by comparing the coronal suture thickness after loading to the control (not loaded) coronal suture thickness. Even with the higher increase in the coronal suture thickness observed, the differences between ex vivo static and in vivo dynamic loading coronal suture thicknesses are not statistically significant and the ex vivo static loading strain estimated in this work is likely a good approximation of the strains experienced during in vivo dynamic loading*.*

Ex vivo strain analysis based on the CT data and the custom code developed in this study provided a full 3D strain analysis of the strain level across the coronal suture at P7 in WT. The estimations aligned with the excepted ranges based on the 2D suture thickness analysis. The 3D analysis also provided an overview of the impact of loading across all calvarial sutures. This provides a more holistic understanding of the impact of the loading across all calvarial sutures in 3D. For example, it highlights that unilateral loading of the frontal bone also induced a relatively high level of strain in the contralateral coronal sutures (e.g. 0.73 ± 0.08 vs. 0.49 ± 0.07 von Mises strain at P7—Fig. 7c). This can explain the observation of bilateral coronal suture patency in some of the MT mice in the study of Moazen et al. (2022). Nonetheless, given that the coronal sutures were at least partially fused at P7, it was not possible to estimate the level of strain induced at the coronal suture in the MT mice at any of the investigated ages. This suture likely experiences a significantly lower level of strain as opposed to the WT mice at the same age, in line with the finding of a lower level of strain in the sagittal suture in the MT compared to the WT at all considered ages. The fused parts of the suture limit the relative movement between the frontal and parietal bones, contributing to the reduction in strain.

A significant decrease in the sagittal suture strain was observed between the P7 and P14 specimens in WT and MT animals. This may be due to the fact that the metopic suture is patent at P7 but closed at P14. This couples the displacement of the frontal bones, and in turn, restricts relative displacement between the two parietal bones. The effect is likely magnified in the MT as, in addition to the metopic, the coronal sutures undergo fusion at this time (Perlyn et al. 2006; Martínez-Abadías et al. 2013; Liu et al. 2013) leading to further limitations in the relative displacement between the two parietal sides. Concurrently, the patent coronal sutures in the WT likely permit increased relative displacement of the two parietal bones leading to a higher sagittal strain compared to the MT at P14. Note, lower average von Mises and first principal strains were observed in the sagittal suture than in either of the coronal sutures at P14, this was statistically significant (see Fig. 7).

There remains a question regarding the level of strain experienced by the coronal suture in the MT mice. This requires further investigation, and computational models based on the finite element method can be a powerful tool. These models can be used to virtually segment this suture and estimate the level of strain experienced by it in the MT mice. Indeed, the data obtained in this study set a strong foundation for future validation of such studies. Our understanding of this structure’s mechanical response to external loading is currently limited. Future in silico models validated by the results presented here will be used to capture these effects.

This study provided important information on the level of strain induced at P7 in both WT and MT mice. Nonetheless, a continuation of this work requires the estimated strain levels at P14 and P21 to be compared to the continuously loaded animals. This would include the possible tissue reorganisation that might have occurred across all sutures in response to the induced in vivo (daily) dynamic mechanical loading from P7 to the aforementioned ages. This can be addressed experimentally or potentially using computer simulations, modelling the tissue reorganisation. (Marghoub et al. 2019; Dolack et al. 2020; Cross et al. 2022).

A potential explanation for the therapeutic effect observed by Moazen et al. (2022) may be that the consecutive cyclic loading disarticulates the frontal and parietal bones as they undergo fusion. The newly formed bone in the sutural space may be a mechanical weak point of the system, and continuous application of force may prevent fusion from progressing. While this was not observed directly during static loading, the hypothesis is strengthened by the observed permanent change in the gap between the frontal and parietal bones observed after cyclic loading in P7 WT mice. Further examination of the coronal suture structure for the fully treated animals is required to understand the underlying mechanism.

The loading parameters considered in this study were either directly (dynamic loading) or indirectly (static loading) based on the parameters that showed an effect on the suture patency during the continuous in vivo loading treatment (Moazen et al. 2022). Consequently, a significant limitation of this work is that the loading parameter space has been left unexplored. Further in*/*ex vivo work will shed light on the effects of varying loading parameters. However, the number of animals required to explore different loading frequencies, forces and timeframes is likely prohibitive. In silico models informed by the mechanical responses provided in this work may be a more cost-effective and ethical approach.

In summary, this study provided insight into the level of displacement and mechanical strain induced during and after both in vivo and ex vivo dynamic loading and ex vivo static loading. Future in silico studies based on the findings presented in this paper can help develop this treatment and help us better understand the complex biomechanical nature of external craniofacial loading.

### Supplementary Information

Below is the link to the electronic supplementary material.

Supplementary file1 (DOCX 5124 kb)
