# Supplementary material for: Multiscale mechanical characterisation of the craniofacial system under external forces
Source: Biomech Model Mechanobiol. 2024 Jan 13;23(2):675–85. doi: 10.1007/s10237-023-01799-y (PMC10963580; doi:10.1007/s10237-023-01799-y)
Supplement: Supplementary file 1 — Supplementary file1 (DOCX 5124 kb) [file 10237_2023_1799_MOESM1_ESM.docx]

**Supplementary materials**

Supplement 1

During CT scanning, the specimen should move as little as possible. However, a constant static load applied to the skull led to significant soft tissue relaxation. Fig. S1 shows the effect of soft tissue relaxation. Here, the loading tip displacement was quantified while applying a constant force of 0.1N to a P7 skull over 180min.

The P7 WT animals were found to show the highest soft tissue relaxation among the considered mice in this study. Hence, the scanning protocol for all specimens was based on these results. The movement can be seen to be mostly stabilised after 120min. Following these findings, all the animals were scanned after 120mins of the static loading.


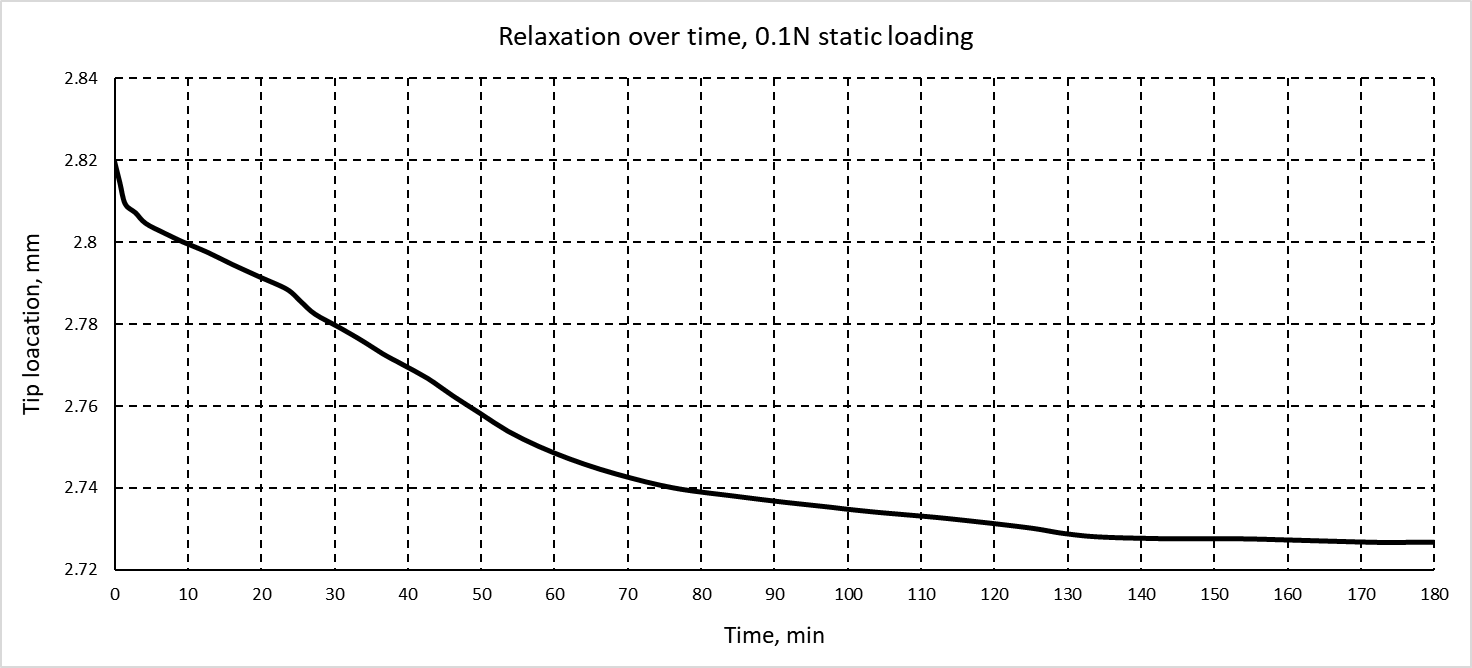


Figure S1. Relaxation as measured by the position of the loading tip for a P7 WT specimen during static loading.

Supplement 2

To build confidence in the strain estimation produced by the developed code, two simplified geometries shown in Fig S2a and b were modelled using commercial finite element software (ANSYS, Pennsylvania, USA) and the code developed here. Mesh independence was carried out on both models in both tools. In brief, mesh independence was examined for both geometries quantitatively and a qualitative analysis of the strain contours in the specimen-specific geometry was produced.

The material properties for the FE analysis were adjusted so the 0.1N load would produce an average von Mises strain of 1 in the suture. This was chosen to capture roughly the same level of displacement observed in the real system during static loading.

When comparing the mesh dependence in Fig S2c for the idealised geometry. It was clear that the FE results were affected by the number of elements in terms of the maximum von Mises strain; however, the difference between the estimated (DSC) and calculated values remained stable, indicating that this had little effect on the approaches ability to capture the deformation patterns.

A comparable pattern was observed in the specimen-specific geometry in Fig S2d. Additionally, it was noted that the average remained stable for both FE and DSC in the simplified geometry. In contrast, a slight decrease in average strain was observed for both FE and DSC results in the animal-specific geometry.

Fig S2d shows the approach’s capabilities in capturing strain patterns in the calvarial sutures for the specimen-specific geometries. Namely, it can be seen that the two strain contours were extremely similar, with slight underestimation in the DSC results. The average von Mises strain across the coronal sutures was underestimated by 3.8% for the 72, 132 and 274 thousand element cases, with the underestimation decreasing to 1.8% for the 1729 thousand element case.

A case with no strain was considered. Here the undeformed surfaces were remeshed to obtain an imaginary “deformed” surface. This surface was compared to the original surface where in fact both surfaces were undeformed surfaces with different surface meshes. The code developed here estimated an average von Mises strain of 0.0117 (that should have been zero). Nonetheless, this represented 1-4% of the measured strains in the sutures of WT animals.

The bone strains however could not be investigated with this approach as the relative displacement within the bone is small and the large elements used to align the undeformed surface to the deformed surface do not allow for the required level of accuracy. As the focus of this work was on the mechanical response of the sutures further refinement to the algorithm used was deemed unnecessary in light of the level of under/overestimation of strain results for the sutures presented in Fig S2d. The method used at best could only estimate the surface strains of the bones.


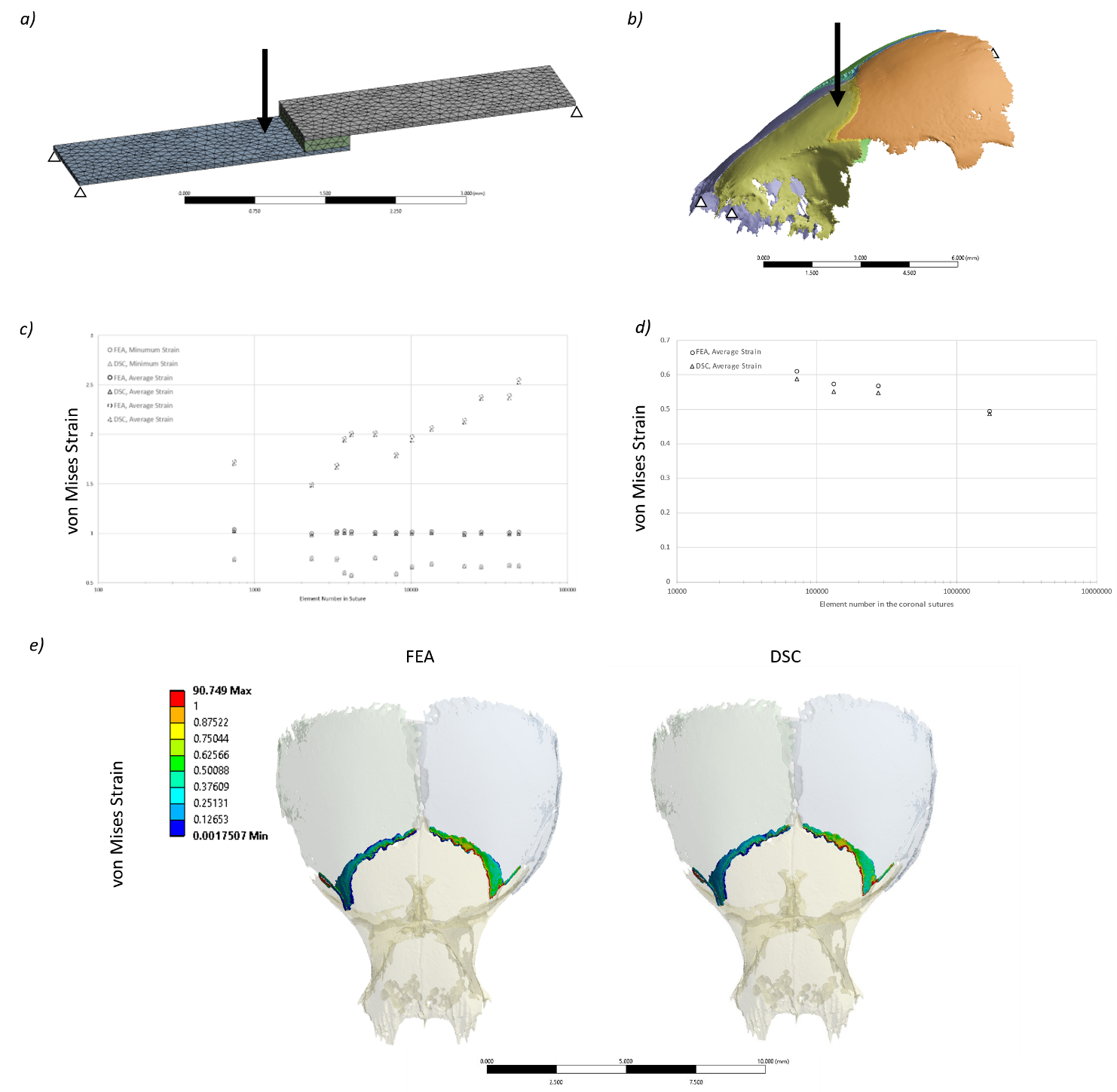


Figure S2. Strain estimation approach sensitivity summary. a) Idealised suture geometry, b) specimen-specific cranial geometry, c) mesh dependence results for the idealised geometry, d) mesh dependence results for the specimen-specific geometry, e)comparison of FEA and DSC strain contours for the coronal sutures.

Supplement 3


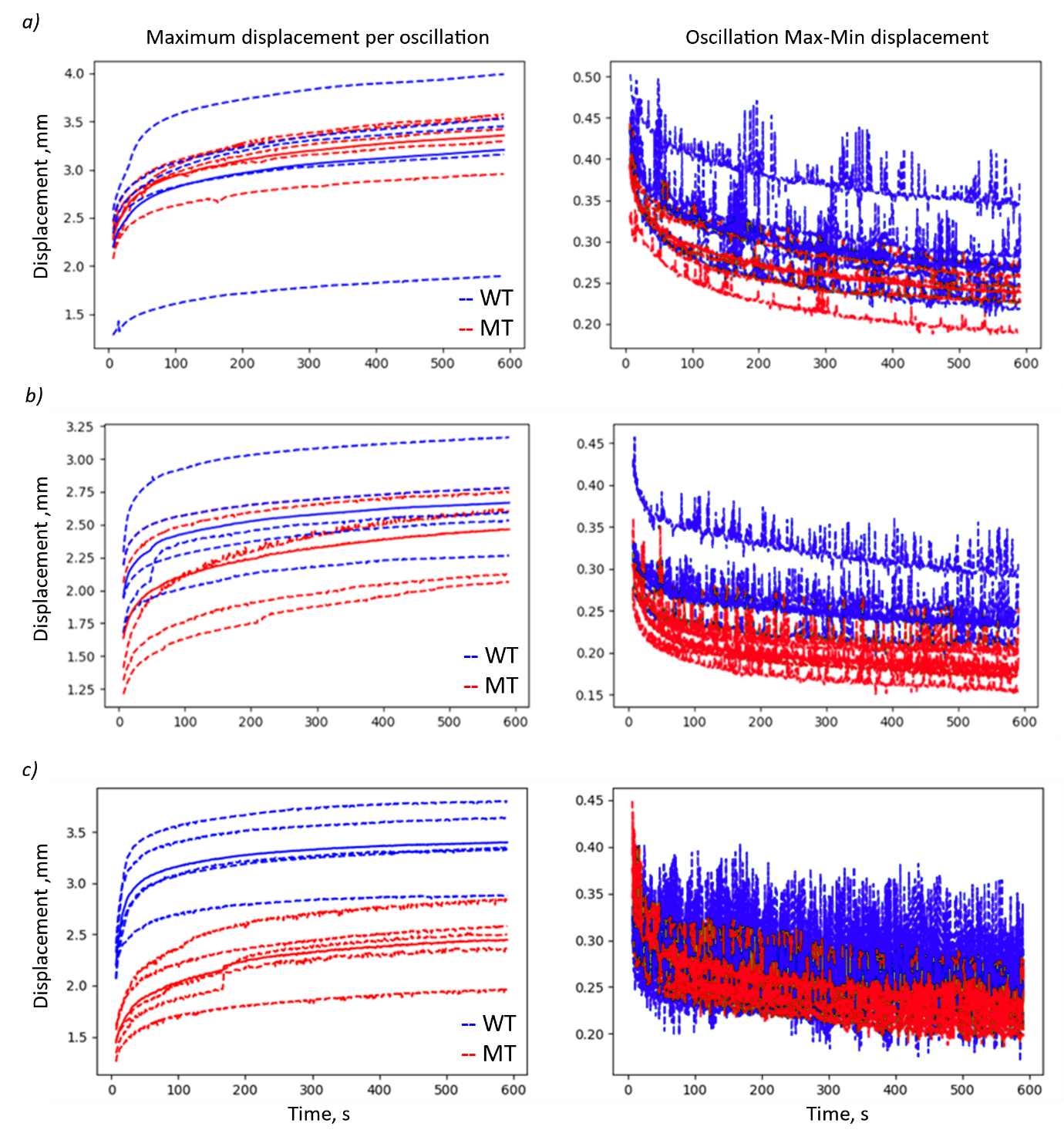


Figure S3. Maximum displacement per oscillation and oscillation Max-Min displacement throughout the 10 minutes of loading at 1Hz and 0.1N. The position was sampled every millisecond. a) P7, b) P14 and c) P21. Dashed lines indicate each specimen, while the solid line indicates the average.

Supplement 4

The strain results indicated a slightly higher absolute value of the first principle strain compared to the third principle strain on the left side coronal suture and the opposite on the pattern on the right side. This may suggest that the left coronal suture experiences more tension than compression, while the opposite is true for the right coronal suture.

To further investigate this, a deeper look into the first and third principle strains is presented in Fig S4 for the WT specimens. The higher third principal strain is evident in the P7 right-side results. While two specimens show this pattern, one shows the opposite pattern. Thus, making it hard to unravel the strain polarity patterns. Further investigation with a larger number of animals should be conducted to confirm the observations made here regarding whether the sutures are in tension or compression.


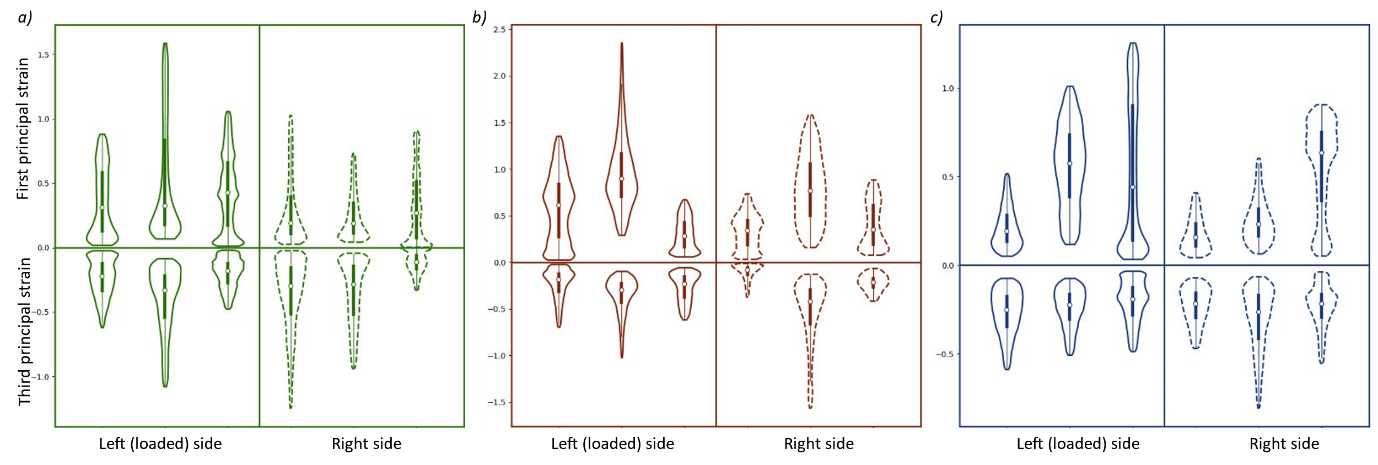


Figure S4. First and third principal strains across both coronal sutures for wild-type specimens at P7, P14, and P21 during ex vivo static loading. This plot presents the distribution of element strains in each specimen individually. A circle indicates the average, bar indicates the quartiles and a line indicates the distribution. A solid line is used for the left side (loaded), and a dashed line is used for the right side. a) P7, b) P14 and c) P21.
